# Whole-Genome Resequencing of Xiangxi Cattle Identifies Genomic Diversity and Selection Signatures

**DOI:** 10.3389/fgene.2022.816379

**Published:** 2022-05-27

**Authors:** Xiaoyu Luo, Jianbo Li, Chentong Xiao, Luyang Sun, Weixuan Xiang, Ningbo Chen, Chuzhao Lei, Hong Lei, Yun Long, Ting Long, Quji Suolang, Kangle Yi

**Affiliations:** ^1^ Hunan Institute of Animal and Veterinary Science, Changsha, China; ^2^ Key Laboratory of Animal Genetics, Breeding and Reproduction of Shaanxi Province, College of Animal Science and Technology, Northwest A&F University, Xianyang, China; ^3^ Xiangxi Cattle Engineering Technology Center of Hunan Province, Huayuan, China; ^4^ School of Life Science, University of Bristol, Bristol, United Kingdom; ^5^ Hunan De Nong Animal Husbandry Group Co. Ltd., Huayuan, China; ^6^ Institute of Animal Science, Tibet Academy of Agricultural and Animal Husbandry Science, Lhasa, China

**Keywords:** Xiangxi cattle, genetic diversity, selection signatures, DNAJC8, whole genome analysis

## Abstract

Understanding the genetic diversity in Xiangxi cattle may facilitate our efforts toward further breeding programs. Here we compared 23 Xiangxi cattle with 78 published genomes of 6 worldwide representative breeds to characterize the genomic variations of Xiangxi cattle. Based on clustering models in population structure analysis, we displayed that Xiangxi cattle had a mutual genome ancestor with Chinese indicine, Indian indicine, and East Asian taurine. Population genetic diversity was analyzed by four methods (nucleotide diversity, inbreeding coefficient, linkage disequilibrium decay and runs of homozygosity), and we found that Xiangxi cattle had higher genomic diversity and weaker artificial selection than commercial breed cattle. Using four testing methods (θπ, CLR, *F*_ST_, and XP-EHH), we explored positive selection regions harboring genes in Xiangxi cattle, which were related to reproduction, growth, meat quality, heat tolerance, and immune response. Our findings revealed the extent of sequence variation in Xiangxi cattle at the genome-wide level. All of our fruitful results can bring about a valuable genomic resource for genetic studies and breed protection in the future.

## Introduction

One of the most economically important breeds of livestock is domestic cattle, providing human beings with basic resources, such as milk, beef, draft energy, and so on. They can be artificially classified into two subspecies: humpless taurine (*Bos taurus*) and humped indicine (*Bos indicus*) (Loftus et al., 1994), which are hybrid with each other. Recently, the research shows that domestic cattle can be categorized into five groups worldwide: European taurine, Eurasian taurine, East Asian taurine, Chinese indicine, and Indian indicine (Chen et al., 2018). As a significant part of cattle variety resources, Chinese native cattle have abundant genetic diversity. Xiangxi cattle, a representative breed of cattle in southwestern China, is one of the breeds recognized by China and renowned abroad, which belongs to the beef-serving type (Supplementary Note). Xiangxi cattle is highly valued because of its strong immunity, excellent meat quality, high service endurance and heat resistance capacity (Wang et al., 2012; Long and Ma, 2009; Zhang et al., 2011). Previous research on genetic diversity, population polymorphism, and hybridization adopted methods, for instance, autosomal, Y chromosome or mitochondrial data (Cai et al., 2019; Luoreng et al., 2014; Yao et al., 2017) to demonstrate that the Xiangxi cattle originated from *Bos taurus* X *Bos indicus*.

With the application of whole-genome sequencing, it has become possible to study the genetic diversity of various breeds on the group size (Daetwyler et al., 2014). Since the implementation of the “The 1,000 Bull Genomes” project, whole-genome sequencing has been used to investigate the world’s commercial breeds (Daetwyler et al., 2014; Stothard et al., 2011) and indigenous breeds (Kim et al., 2017; Lee et al., 2013), providing precious genome resources for future molecular breeding and genetic improvement. As a hot research methodology, the whole-genome sequencing has been progressively applied to Chinese native cattle such as Qinchuan cattle, Jiaxian Red cattle, and Yanbian cattle (Choi et al., 2015; Mei et al., 2018; Xia et al., 2021).

Considering that the entire genome variation of Xiangxi cattle was largely unexplored, we performed whole-genome sequencing on 23 Xiangxi cattle. SNPs were identified in Xiangxi cattle by basing on *Bos taurus* reference genome assembly (ARS-UCD1.2). Then SNPs of Xiangxi cattle made a comparison with those of commercial and native breeds previously collected from around the world. Finally, by scanning the whole genome of Xiangxi cattle, the selective sweeping results were determined. Our analyses fully described the genomic diversity and population structure, revealed the possible signs of natural and artificial selection, as well as provided new insights for breeding Xiangxi cattle.

## Methods

### Samples, DNA Extraction, and Sequencing

Ear tissue samples of Xiangxi cattle (*n* = 23) were collected from the Xiangxi cattle engineering technology center of Hunan Province, Huayuan, China. Genomic DNA of the ear tissue samples was extracted using a standard phenol/chloroform-based protocol. The DNA library was constructed for each sample (500 bp insert size). Sequencing via Illumina NovaSeq 6000 with 2 × 150 bp model at Novogene Bioinformatics Institute, Beijing, China, and 150 bp paired-end sequence data were generated.

Furthermore, we obtained sequence data of 78 cattle, including 25 European cattle (Hereford-14, Jersey-11), 15 Hanwoo, 31 Chinese native cattle (Wannan-5, Guangfeng-4, Ji’an-4, Leiqiong-3, Jinjiang-3, Qinchuan-12), and 7 India-Pakistan cattle (Tharparkar-1, Sahiwal-1, Hariana-1, Nelore-1, Gir-2 and unknown-1) publicly. In total, 101 whole genomes of cattle were used for the subsequent analysis.

### Reads Mapping and SNP Calling

The clean reads were mapped onto the *Bos taurus* reference genome assembly ARS-UCD1.2 using BWA-MEM (0.7.13-r1126) with default parameters (Li and Durbin, 2009). After mapping, the single nucleotide polymorphisms (SNPs) were detected by using Samtools (Li et al., 2009), Picard tools (http://broadinstitute.github.io/picard), and Genome Analysis Toolkit (GATK, version 3.6–0-g89b7209). All SNPs were filtered using the module “Variant Filtration” of GATK to obtain high-quality SNPs. By using the ANNOVAR(Wang et al., 2010), SNPs were annotated based on the latest reference assembly (ARS-UCD1.2).

### Population Genetics Analysis

After pruning in PLINK with the parameter (--indep-pair-wise 50 5 0.2), a set of SNPs were generated for the following analyses. An unrooted neighbor-joining (NJ) was constructed based on the matrix of pairwise genetic distances using MEGA v7.0 (Kumar et al., 2016) and iTOL v5 (Letunic and Bork, 2021). Principal component analysis (PCA) was performed using the smartPCA of the EIGENSOFT v5.0 package (Patterson et al., 2006). Population structure analysis was assessed with genetic clusters K ranging from 2 to 7 using the ADMIXTURE v1.3 (Alexander and Large, 2011).

Linkage disequilibrium (LD) decay was calculated using PopLDdecay (Zhang et al., 2019) with default parameters. By using the VCFtools (Danecek et al., 2011), we respectively calculated the inbreeding coefficient (--het) and the Nucleotide diversity (π). Additionally, we identified the runs of homozygosity (ROHs) using the--homozyg option implemented in the PLINK. The number and length of ROH for each breed were calculated and divided into four categories (0.5–1 Mb, 1–2 Mb, 2–4 Mb, >4 Mb). The plot as mentioned above was depicted using the R script (http://www.r-project.org).

### Selective Sweep Test

To identify the selective sweep regions in Xiangxi cattle, two different statistics were used, including the nucleotide diversity (θπ) with 50 kb sliding window and 20 kb step in VCFtools (Danecek et al., 2011) and the composite likelihood ratio (CLR) with 50 kb windows in SweepFinder2 (DeGiorgio et al., 2016). We took the top 1% of the overlapping genes obtained by the two above methods.

For comparison in Xiangxi cattle and Qinchuan cattle, we separately calculated fixation index (*F*_ST_) and cross-population extended haplotype homozygosity (XP-EHH). We estimated the *F*_ST_ values using VCFtools (50 kb windows with 20 kb step) and the XP-EHH using selscan v1.1 (Szpiech et al., 2014) with default settings. It is noteworthy that the normalized XP-EHH score was tested about each 50-kb region in the XP-EHH statistic. Significant genomic regions were identified (*p* < 0.005). In order to make the results more reliable, we used two or more methods to determine that the overlapping regions had been used as candidate regions. We calculated the Tajima’s D statistic to consolidate our results by using VCFtools.

Moreover, KOBAS 3.0 (http://kobas.cbi.pku.edu.cn/) was used to understand the function and complex pathways of candidate genes, including the Kyoto Encyclopaedia of Genes and Genomes (KEGG) and Gene Ontology (GO) in the study (corrected *p*-value < 0.05).

## Results

### Sequencing and Identification of Single Nucleotide Polymorphisms

Using BWA-MEM software, the clean reads were aligned with the *B. taurus* reference genome (ARS-UCD1.2), resulting in a total of ∼4.7 billion reads and ∼10.9 × coverage each (Supplementary Table S1). Xiangxi cattle were jointly analyzed with the public genomic data of five “core” populations (Chen et al., 2018; Xia et al., 2021)and Qinchuan cattle (native cattle in North-central China). Five “core” populations: European taurine (Hereford), Eurasian taurine (Jersey), East Asian taurine (Hanwoo), Chinese indicine (Leiqiong, Guangfeng, Ji’an, Jinjiang and Wannan), and Indian indicine (Tharparkar, Nelore, Sahiwal, Hariana and Gir) (Supplementary Table S2).

Xiangxi cattle have been discovered with 40573969 biallelic SNPs. The functional annotation of the polymorphic loci displayed that the vast majority of SNPs existed in the intergenic region (60.1%) or intron region (38.2%). Exon accounted for 0.7% of the total SNPs, including 112572 nonsynonymous and 179112 synonymous SNPs (Supplementary Figure S1). In addition, 0.9% of the SNPs were detected in an untranslated region (UTR), and the remaining 0.1% in splice. Xiangxi cattle was found to have the largest number of SNPs which was consistent with Chinese indicine on the whole, whereas Qinchuan was only second to them (Supplementary Table S3).

### Population Structure and Characterization

**FIGURE 1:**
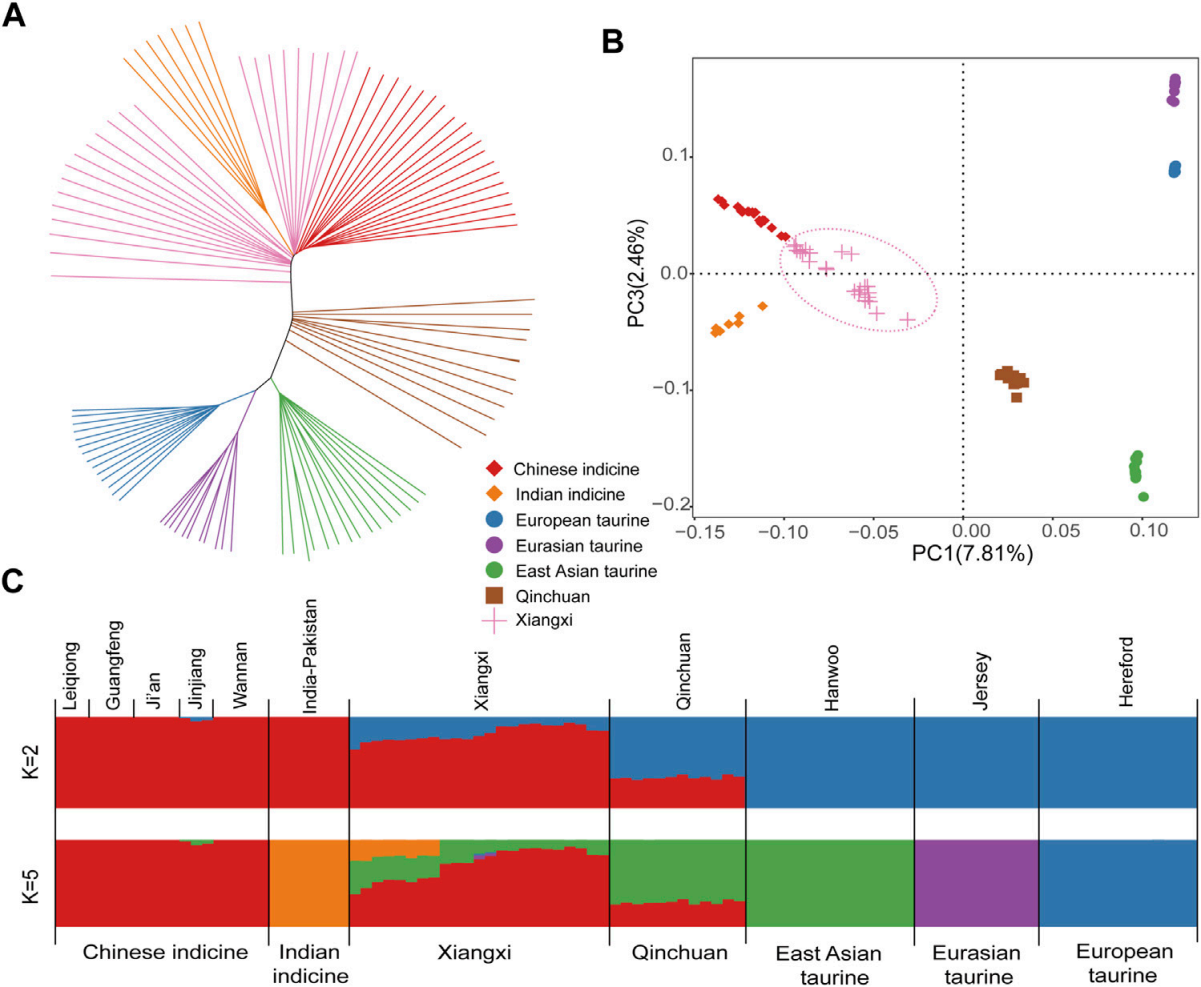
Population genetic analysis of Xiangxi cattle. **(A)** The Neighbor-joining tree of relationships among 7 populations **(B)** The principal component analysis of cattle with PC1 against PC3 **(C)** genetic structure of cattle using ADMIXTURE with K = 2 and K = 5.

Based on the study of autosomal SNPs, we discussed the phylogenetic relationships among 101 samples from 7 populations (Figure 1). The NJ (neighbor-joining) tree showed that all “core” cattle populations form separate clusters. Qinchuan cattle are located between taurine cattle and indicine cattle (Mei et al., 2018), while Xiangxi cattle are aggregated near Chinese indicine and Indian indicine (Figure 1A). Principal component analysis (PCA) provides similar results to the above conclusions (Figure 1B).

We estimated the ancestral populations of all cattle samples by using clustering models. When K = 2, the lineage of cattle can be fundamentally distinguished from that of taurine and indicine cattle. When K = 5, five “core” cattle herds were separated, while Xiangxi cattle exhibited mixed phenomena (Figure 1C). The result displayed that it had a mutual genome ancestor with Chinese indicine, Indian indicine, and East Asian taurine. Moreover, a more dramatic genetic influence of Chinese indicine than the others was shown in the results.

### Population Genetic Diversity

**FIGURE 2:**
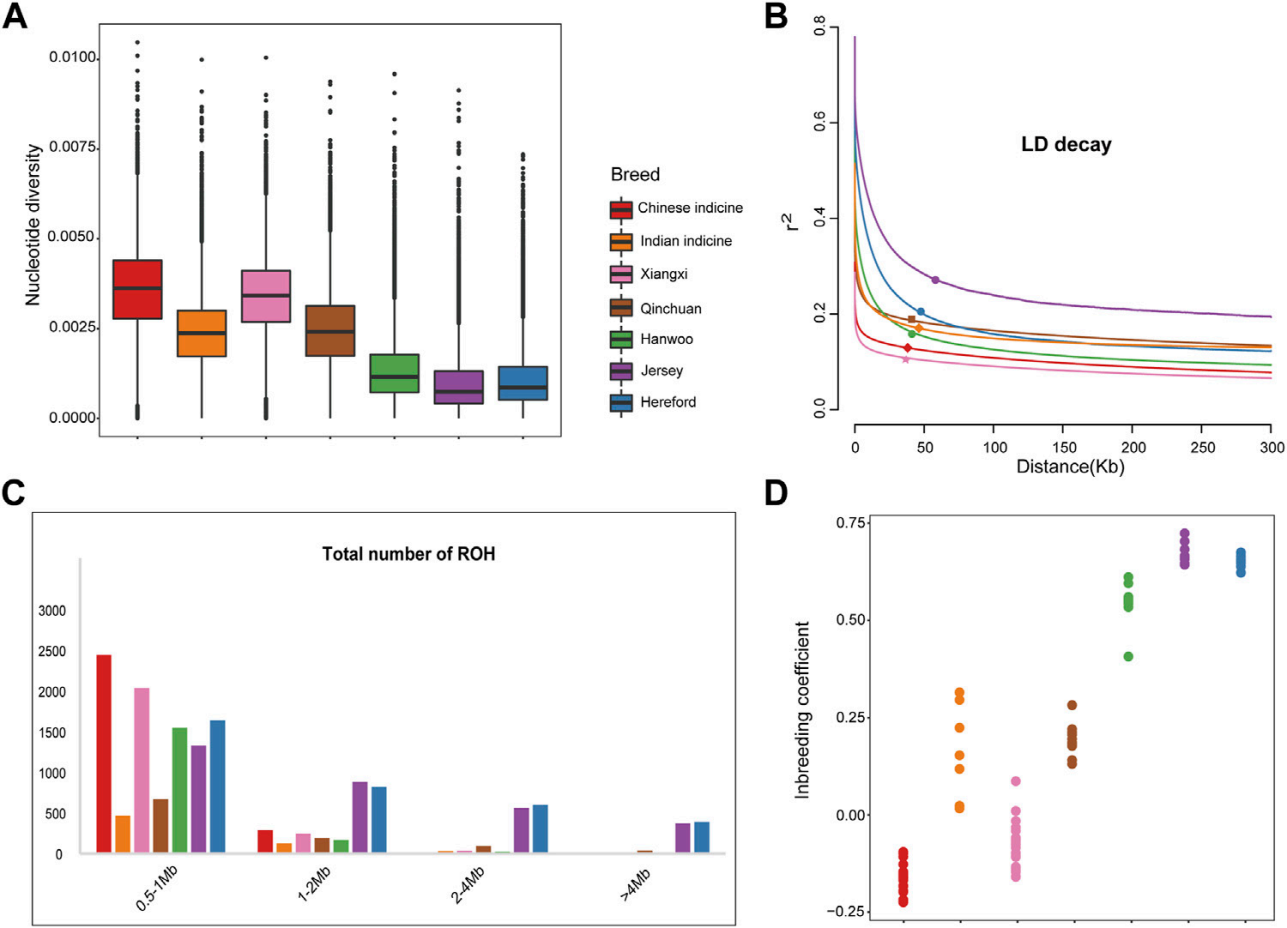
Genetic diversity among 101 samples from 7 populations. **(A)** Box plots of the nucleotide diversity for each group. The points which were on the outside the of whiskers showed outliers **(B)** Decay of linkage disequilibrium on cattle autosomes estimated from each breed. **(C)** The estimation of total number of ROH for each group **(D)** Inbreeding coefficient for each individual.

As shown in Figure 2A, the nucleotide diversity of Xiangxi cattle (0.00339) was second only to Chinese indicine (0.00357) and close to Qinchuan cattle (0.00247) and Indian indicine (0.00238), while that of Eurasian taurine (0.00095) was the lowest. The results of the linkage disequilibrium analysis agreed with these findings. The lowest LD level was found at short distances in Xiangxi cattle, followed by Chinese indicine, and the Eurasian taurine (Jersey) showed a higher LD level (Figure 2B).

Meanwhile, the ROH of each breed was divided by length (0.5–1 Mb, 1–2 Mb, 2–4 Mb and >4 Mb). In order of the number of ROH, it demonstrated that the European taurine took the largest number. By contrast, the number of the Indian indicine is lowest, that of the Chinese Crossbreed and Chinese indicine are in the middle respectively (Figure 2C). The Xiangxi cattle we were concerned with showed more extensive amounts of short/medium ROH (0.5–2 Mb) but there were no long ROH (2–4 Mb). The number of ROH in European taurine cattle is much larger than in Xiangxi cattle due to high breeding. It is noteworthy that highly selective breeding may be at risk of inbreeding depression. As expected, the inbreeding coefficient also proved these findings (Figure 2D).

### Genome-Wide Selective Sweep Test

**FIGURE 3:**
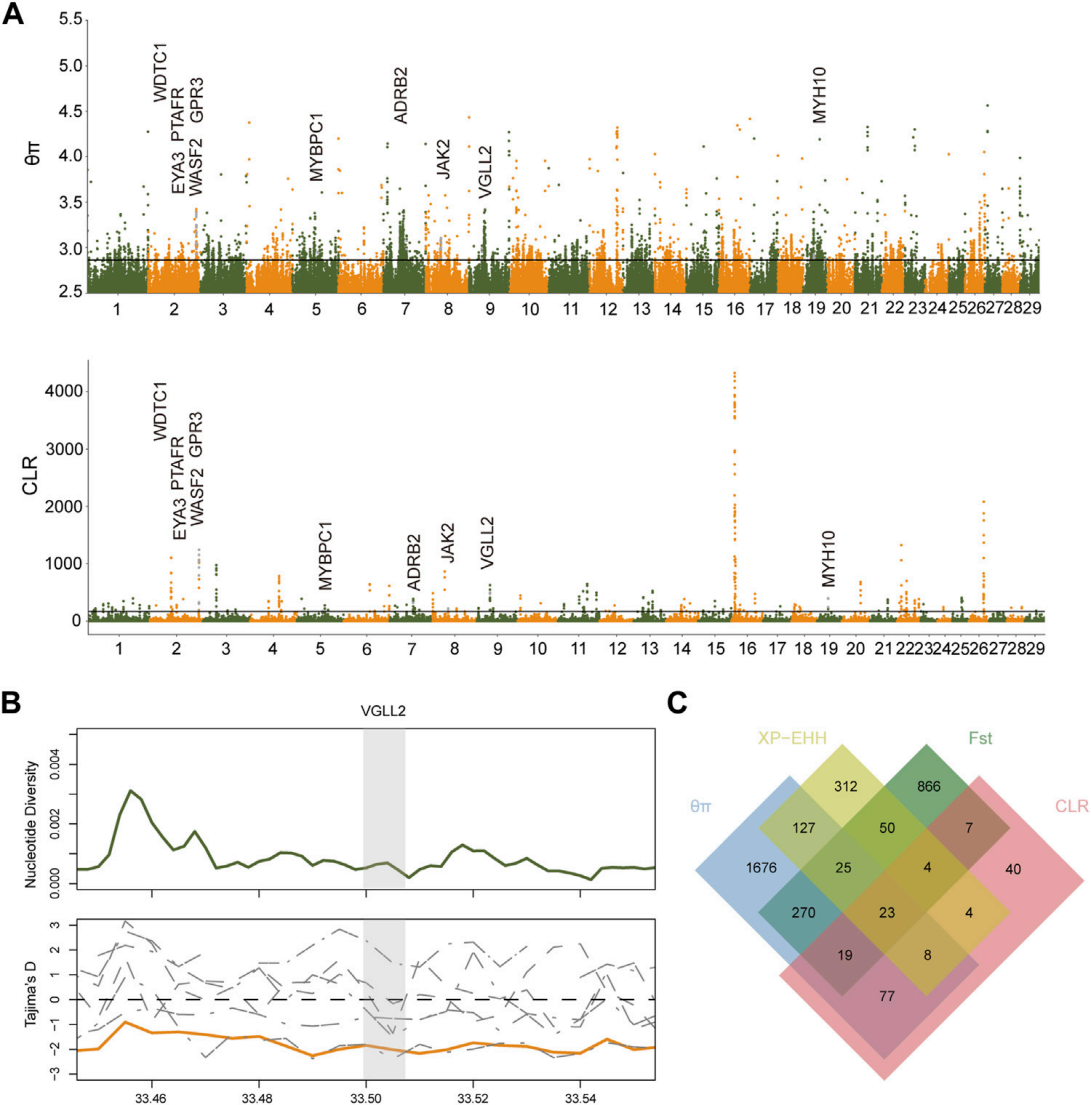
The signatures of positive selection in Xiangxi cattle. **(A)** Manhattan plot of selective sweeps by θπ and CLR methods **(B)** Nucleotide diversity of *VGLL2* gene region in Xiangxi cattle. and Tajima’s D value in each group (orange line in Xiangxi cattle). **(C)** Number of candidate genes identified in Xiangxi cattle by the four methods (θπ, CLR, *F*_ST_ and XP-EHH) listed in each of the Venn diagram components.

For the sake of screening out the associated genetic variation, the nucleotide diversity analysis (θπ) and the composite likelihood ratio (CLR) were used to explore the selected genomic regions in Xiangxi cattle (Supplementary Tables S4, S5) (Figure 3). We found that partial genome regions in Xiangxi cattle might have been selected during domestication. A total of 2225 (θπ) and 182(CLR) genes were identified. Among them, 127 genes were overlapped which were considered to be candidate genes (Supplementary Table S6). The annotations of candidate genes revealed the functions that may be associated with economic traits, including reproduction (*KHDRBS2*, *GOLGA4*, *BBX*, *BANF2*, *INSL6*) (Cai et al., 2017; Guo et al., 2018; Ortega et al., 2016; Pini et al., 2020; Wang et al., 2016), growth (*RALGAPA1*, *NCK1*, *VGLL2, FGFR3*) (Aryal et al., 2015; Honda et al., 2017; Sun et al., 2020; Wen et al., 2016), meat quality (*PLIN4*) (Pena et al., 2019), and somatotype (*SYN3*) (An et al., 2019). We also obtained genes (*ELANE, AZU1*) (Cui et al., 2021; Zhang et al., 2021) related to immune response, which may result from long-term natural selection.

**FIGURE 4:**
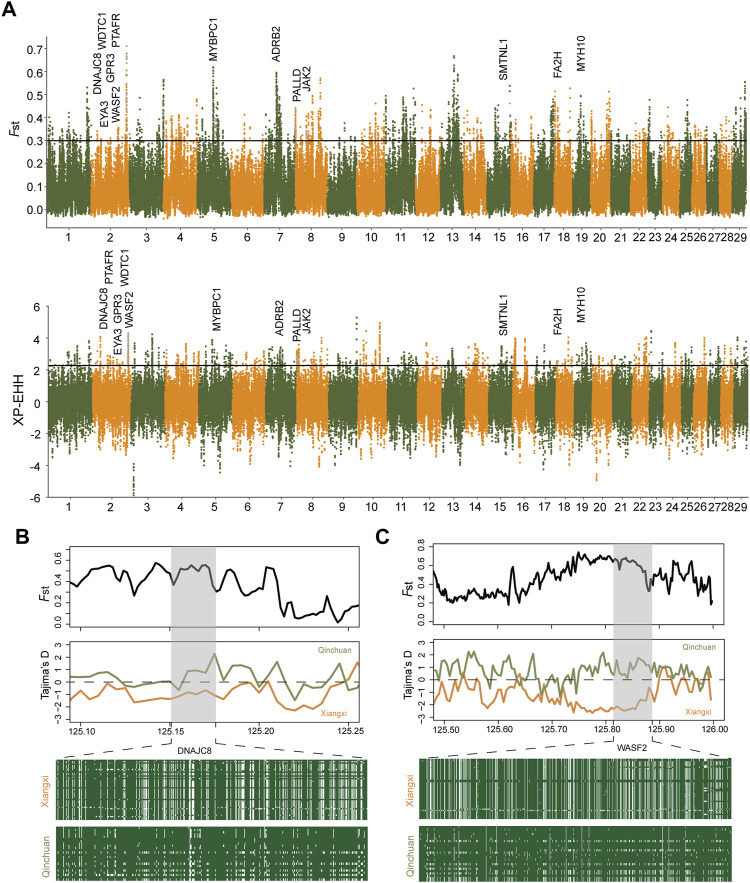
Selective signals between Xiangxi cattle and Qinchuan cattle. **(A)** Manhattan plot of selective sweeps by *F*_ST_ and XP-EHH methods. *F*_ST_ and Tajima’s D plots of *DNAJC8* gene **(B)** and *WASF2* gene **(C)**. Haplotype diversity at the example genes **(B,C)**.

**FIGURE 5:**
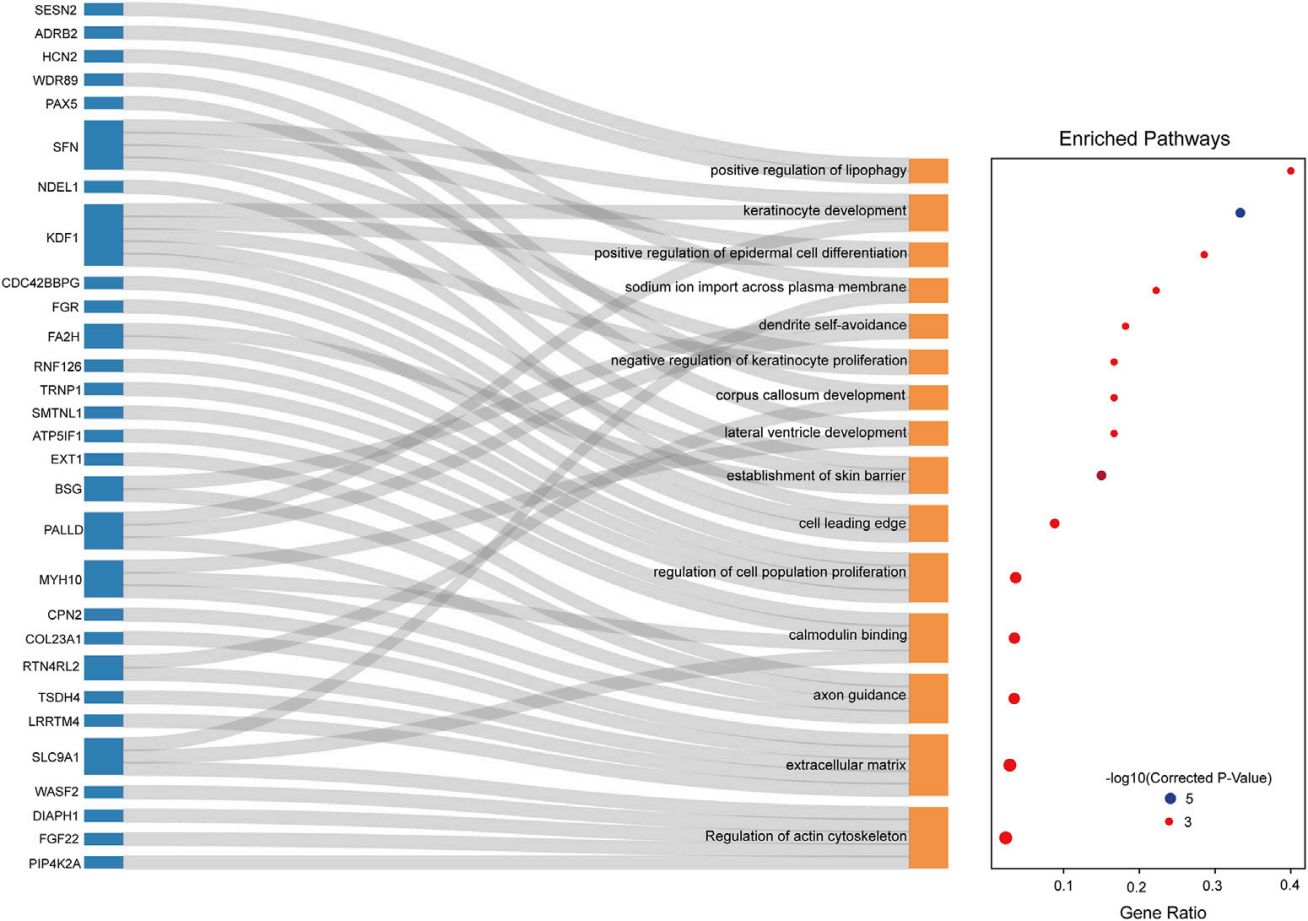
KEGG and GO enrichment analysis of Xiangxi cattle candidate genes overlapped by *F*_ST_ and XP-EHH methods (corrected *p*-value < 0.05).

Furthermore, we implemented two methods, *F*_ST_ (*p* < 0.005, *F*_ST_ ≥ 0.29979) and XP-EHH (*p* < 0.005, XP-EHH≥2.27), to elucidate further the positive selection characteristics between Xiangxi cattle and Qinchuan cattle (Figure 4), which contained 1,264 and 553 hypothetical selection genes, respectively (Supplementary Tables S7, S8). 102 overlapping genes were also tested in both methods (Supplementary Table S9). Among them, regions containing known candidate genes related to heat tolerance (*DNAJC8*) (Li G. et al., 2020) showed intense differentiation signals. As for functional enrichment analysis (Figure 5), the KEGG pathway had only one significant pathway called “Regulation of actin cytoskeleton” (corrected *p*-value < 0.05) and 5 genes were performed (*PIP4K2A*, *FGF22*, *DIAPH1*, *WASF2*, *SLC9A1*), which were related to meat quality, growth efficiency, and the balance of ionic concentrations (Supplementary Table S10). GO terms were enriched, in particular including the terms of skin barrier and epidermal cell differentiation (“establishment of skin barrier, GO:0061436” “positive regulation of epidermal cell differentiation, GO:0045606” “negative regulation of keratinocyte proliferation, GO:0010839”). These several genes (*SFN*, *PALLD*, *KDF1*, *FA2H*) were found in Xiangxi cattle which were associated with the development of sebum, sebaceous glands, and epidermal cells. In addition, we also detected significant GO terms responsible for meat quality (“positive regulation of lipophagy, GO:1904504” “calmodulin binding, GO:0005516”) (Supplementary Table S11) involving relevant genes (*ADRB2*, *SESN2*, *MYH10*, *ATP5IF1*, *SMTNL1*, *SLC9A1*).

Above all, among the above four applied methods, 23 candidate genes were involved in production and immunization traits (Supplementary Table S12). e.g., The *ADRB2* gene was associated with muscle pH (Murani et al., 2013). The *MYH10* gene was discovered to potentially affect the beef quality of the longissimus lumborum muscle following castration (Li et al., 2020b). *WASF2* gene affects cytoskeleton arrangement. The *EYA3* gene was revealed to have a significant correlation with muscle development (Xia et al., 2021). *MYBPC1* genes are well-known marbling-related genes, which were first identified in Japanese Black beef cattle (Li et al., 2020c). The *GPR3* gene was relative to fat development in mice (Godlewski et al., 2015). The *WDTC1* gene was involved in the regulation of fat-related gene transcription (Ducos et al., 2017). PTAFR was also reported to be involved in inflammatory responses in cattle (Trovato et al., 2015; Vanvanhossou et al., 2020; Xerxa et al., 2016). *JAK2* mediates essential signaling events in innate and adaptive immunity (Lukashova et al., 2003).

## Discussion

In order to figure out the population structure and genetic diversity of Xiangxi cattle, we utilized the representative worldwide cattle dataset, making a comparison in a number of contexts and analyses as follows. Ancestor Component Analysis can reflect the communicating extent of genetic information. Xiangxi cattle was complex in the lineage composition which included Chinese indicine (∼73%), East Asian taurine (∼20%), and Indian indicine (∼7%) (Supplementary Figure S2). The heterogeneity of Chinese cattle was induced by different taurine and indicine lineage proportions. Since Xiangxi cattle was situated in the southern regions of China, its ancestry was affected by indicine more than taurine cattle. On the contrary, Qinchuan cattle was situated in North-central China. Using the principal component and neighbor-joining tree analytical methods, we observed an accurate division of seven populations. In particular, Xiangxi cattle were markedly separated into two branches (Figure 1A), which may be caused by the short-term hybridization of foreign commercial cattle, or the self-domesticated Indian zebu which have been migrated from the Indus Valley in the East and entered China from Yunnan Province about three thousand years ago. It may subsequently have an effect on southern Chinese cattle.

In the present study, the genomic variation parameters of the populations all had a similar trend. We ranked all breeds according to their nucleotide diversity (Chinese indicine > Xiangxi cattle > Qinchuan cattle > Indian indicine > taurine cattle), which was consistent with the previous study (Liu et al., 2020). By contrasting the two Chinese breeds, it was found that the genetic diversity of Xiangxi cattle exceeded the Qinchuan cattle, possibly because of the fact that Xiangxi cattle contained more Chinese indicine cattle lineage. The highest genetic diversity observed in Chinese indicine was induced by introgression of other bovines (*Bos javanicus*) (Chen et al., 2018). Similarly, the analysis of SNPs, inbreeding coefficient, and LD decay was highly concurrent with the abundant genetic diversity of Xiangxi cattle. The length and distribution density of ROH revealed that the degree of Xiangxi cattle was obviously less than taurine (Figure 2). Our results showed that Xiangxi cattle had more genetic diversity than taurine cattle due to insufficient breeding. After a long period of intensive breeding, taurine cattle have formed a mature commercial system, whereas the breeding system of Xiangxi cattle was imperfect and has tremendous potential.

Xiangxi cattle is an advantageous resource for the development of animal husbandry because of its excellent characteristics, especially for its disease-resistance and environmental adaptability. To increase the assay efficiency and decrease false positives, four selective sweeping methods were performed on Xiangxi cattle. If the gene was conspicuously detected by at least two methods, then it will be served as an actual candidate gene. Xiangxi cattle is muscular, and have long been used as draft animals. According to θπ and CLR (Figure 3A), the overlapped candidate gene (*VGLL2*) played a vital role in the process of locomotion. The *VGLL2* gene can affect the development of skeletal muscles (Gabriel et al., 2016; Honda et al., 2017,^2019^). Subsequently the positive selection region including the *VGLL2* gene was intensively confirmed by the lower values of Tajima'D and nucleotide diversity analysis in Xiangxi cattle (Figures 3B,C).

The surroundings of Xiangxi cattle are hotter than Qinchuan cattle. In the long-term natural and artificial selection, Xiangxi cattle has adapted to the thermal climate in southern China. Using Qinchuan cattle as the reference population, we calculated *F*_*ST*_ and XP-EHH (Figure 4A). The *DNAJC8* gene, a member of DnaJ Heat Shock Protein Family, was observed to act as an important part of heat stress. Previous studies suggested that *DNAJC8* protected bees from heat stress by regulating heat-inducible and antioxidant genes (Li G. et al., 2020). Heat stress triggers the production of reactive oxygen species (ROS) (Slimen et al., 2014). Previous studies have shown that DnaJ protein plays an important role in protecting antioxidant enzyme activity and removing excess ROS(Kong et al., 2014; Wang et al., 2014). In order to avoid false positives, the verification analysis was carried out on the gene region. *DNAJC8* was located on chromosome 2 of cattle, about 0.025 Mbp. This region showed extremely apparent differentiation and distinct haplotype patterns in two populations. Also, Tajima‘D analysis exhibited that it was significantly lower in Xiangxi cattle (Figure 4B). These results provided forceful evidence that the region containing *DNAJC8* may have a crucial effect on heat resistance in Xiangxi cattle.

In addition, GO terms (e.g., keratinocyte development, establishment of skin barrier, positive regulation of epidermal cell differentiation) were enriched, involving epidermal cell related genes (e.g., *SFN*, *PALLD*, *KDF1*, *FA2H*) (Figure 5). The *SFN* gene can affect the expression of key matrix metalloproteinase (MMPs) in skin fibroblasts, thereby controlling the process of cutaneous wound healing (Kilani et al., 2008; Medina et al., 2007). *FA2H* is essential for the synthesis of HFA-sphingolipids in various organs which is highly expressed in the skin and crucial for the formation of the epidermal barrier (Maier et al., 2011). Xiangxi cattle strengthen the resistance to external toxins and prevent excessive loss of body fluids through the action of keratinocytes, thus adapting to the local heat and humid environment better. A KEGG pathway called “Regulation of actin cytoskeleton” enriched genes (*PIP4K2A*, *FGF22*, *DIAPH1*, *WASF2*, *SLC9A1*) associated with the muscle contraction. *SLC9A1* improves the adjustment capacity of muscle pH value and maintains body endurance (Iaia et al., 2008; Skovgaard et al., 2014). It was worth noting that the *WASF2* gene, which was overlapped in the four selection methods, was a downstream effector molecule that was involved in the signal transduction from tyrosine kinase receptors and small GTPases to actin cytoskeleton and promoted actin filament formation (Miki et al., 1998). The gene can respond to changes of the external environment by reregulating its expression or distribution to further influence the cytoskeleton arrangement (Qian et al., 2009). Besides, *WASF2* was also related to innate immunity, which may be attributed to the pleiotropy of the gene (Liu et al., 2021; Nolz et al., 2006). Compared to Qinchuan cattle, Xiangxi cattle conserved more *WASF2*. The haplotype patterns of the *WASF2* gene were different between the two populations (Figure 4C). Therefore, it was speculated that the *WASF2* gene was selected in Xiangxi cattle. In the past, due to the local geographical environment and harsh production conditions, Xiangxi cattle were mainly selected for draft, resulting in the characteristics of high endurance and strong assault draft. The above genes might be related to these characteristics.

In four selected methods (θπ, CLR, *F*_ST_, and XP-EHH), six genes (*ADRB2*, *WDTC1*, *MYBPC1*, *EYA3*, *MYH10*, *GPR3*) also showed positive selection, which may be related to superior meat quality traits in Xiangxi cattle. Two genes (*PTAFR*, *JAK2*) were related to the disease-resistance of Xiangxi cattle. *PTAFR* gene, platelet activating factor receptor, can regulate inflammatory, smooth-muscle contractile and hypotensive activity (Honda et al., 2002; Marathe et al., 1999). Additionally, *JAK2* is one of the tyrosine kinase family genes related to cytokine receptors, which has a certain correlation with *PTAFR* gene in immune response (Lukashova et al., 2003). Therefore, they were vital for the disease resistance of Xiangxi cattle.

In summary, Xiangxi Cattle is one of the famous local excellent breeds in China with superior traits (disease-resistance, environmental adaptability, meat quality). Our selective sweep identification of Xiangxi cattle may promote the cognition of the hereditary mechanism of potential population characteristics, and hopefully serve as a theoretical reference for the breeding in the future.

## Conclusion

In this study, we used WGS data to study the population structure of Xiangxi cattle, so as to provide the first in-depth study of its gene diversity, phylogenetic relationship, and Selective Sweep Test. Some genes were identified that will not only be helpful to us for a better understanding of the features of Xiangxi cattle, but also further study the characteristics of other native cattle of China. In conclusion, the revelation of the genetic diversity of Xiangxi cattle will establish a sound foundation for conservation breeding in the future.

## Data Availability

The datasets presented in this study can be found in online repositories. The names of the repository/repositories and accession number(s) can be found below: https://www.ncbi.nlm.nih.gov/genbank/, accession number: PRJNA779877.
