# Supplementary material for: Whole-Genome Resequencing of Xiangxi Cattle Identifies Genomic Diversity and Selection Signatures
Source: Front Genet. 2022 May 27;13:816379. doi: 10.3389/fgene.2022.816379 (PMC9196905; doi:10.3389/fgene.2022.816379)
Supplement: Supplementary file 1 [file DataSheet2.pdf]

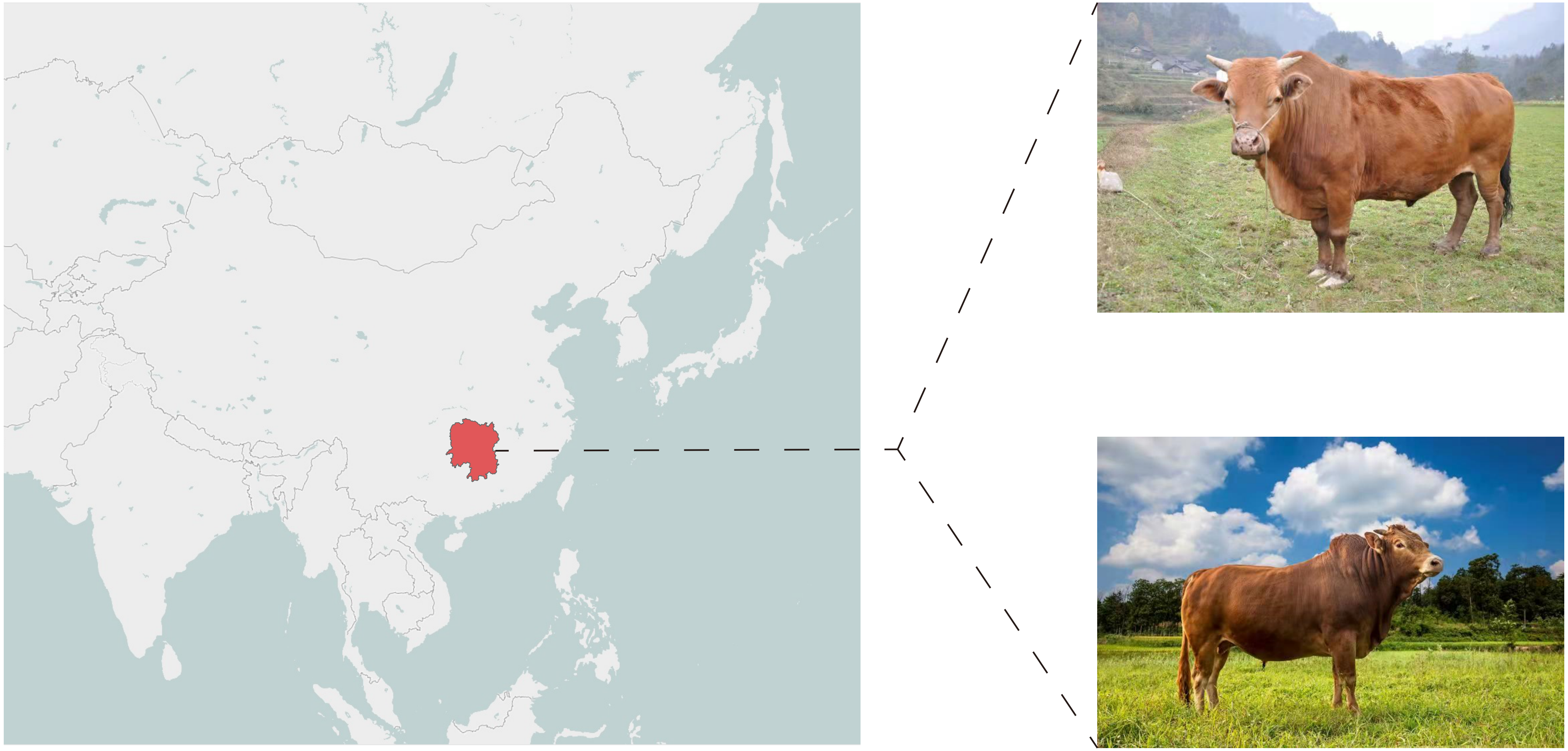

Supplementary Note. Descriptions of the Xiangxi cattle in this study.

Xiangxi cattle that belongs to chinese native cattle (crossbreed between Bos indicus and Bos taurus cattle) are mainly found in the Xiangxi Tujia-Miao autonomous region, Hunan Province, China. In 2006, Xiangxi cattle was listed in the National Resource protection list by the Ministry of Agriculture for its rarity and non-replicability.
