# Supplementary figures and images for: Whole-Genome Resequencing of Xiangxi Cattle Identifies Genomic Diversity and Selection Signatures
Source: Front Genet. 2022 May 27;13:816379. doi: 10.3389/fgene.2022.816379 (PMC9196905; doi:10.3389/fgene.2022.816379)

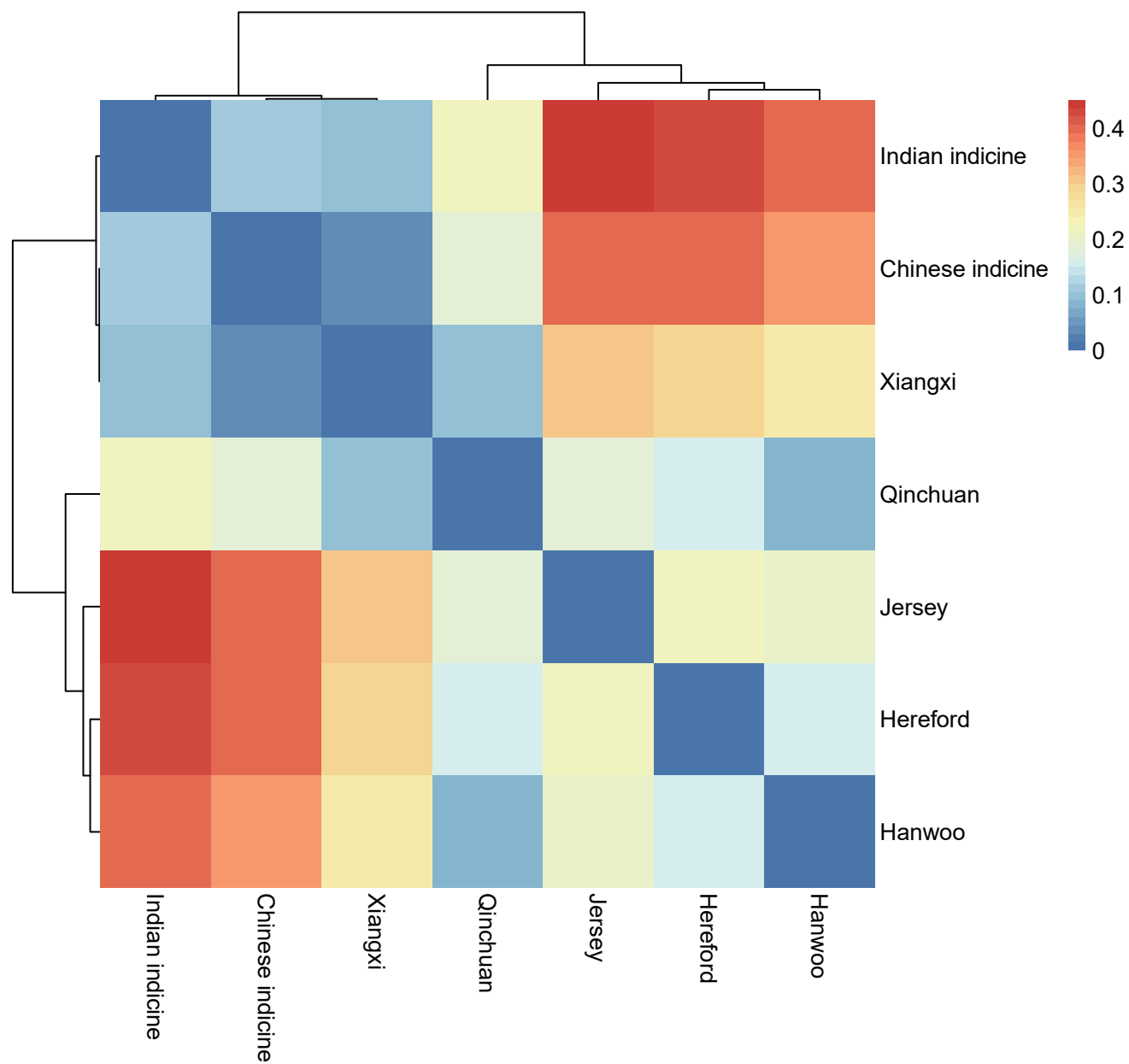

**Supplement Figure S2.** Genetic differentiation matrix among seven breed.

Supplement: Supplementary file 2 [file Image2.pdf]

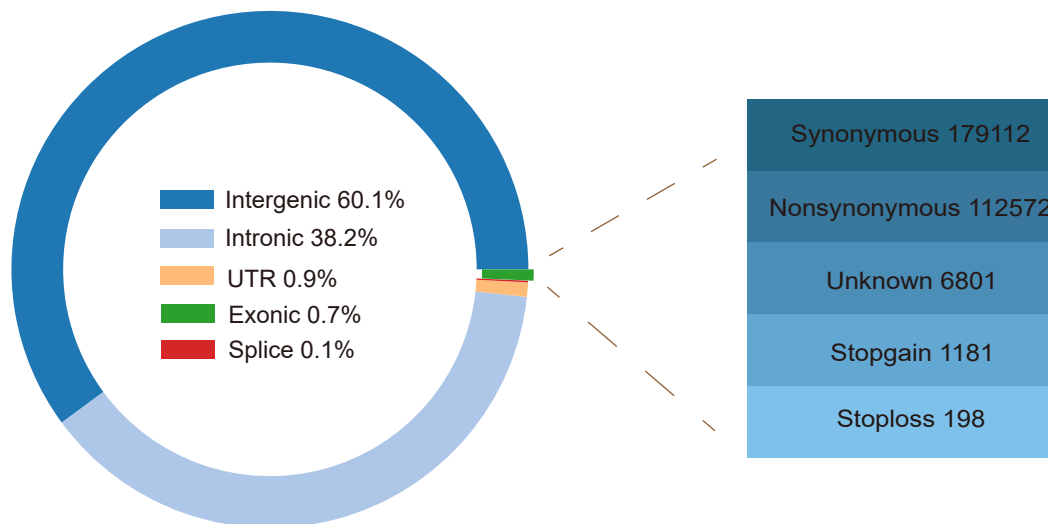

**Supplementary Figure S1.** Functional classification of the detected SNPs in Xiangxi cattle.

Supplement: Supplementary file 3 [file Image1.pdf]
